# Hospital-onset bacteremia and fungemia: examining healthcare-associated infections prevention through a wider lens

**DOI:** 10.1017/ash.2023.486

**Published:** 2023-11-08

**Authors:** Gregory M. Schrank, Graham M. Snyder, Surbhi Leekha

**Affiliations:** 1 Department of Medicine, University of Maryland School of Medicine, Baltimore, MD, USA; 2 Department of Epidemiology and Public Health, University of Maryland School of Medicine, Baltimore, MD, USA; 3 Department of Medicine, University of Pittsburgh School of Medicine, Pittsburgh, PA, USA

## Abstract

A hospital-onset bacteremia and fungemia (HOB) metric will expand hospital surveillance of bloodstream infections beyond current state and provide an opportunity to re-evaluate infection prevention strategies. Here we consider the added value and potential pitfalls of HOB surveillance and present a framework for the standardized assessment of HOB events.

In the enduring effort to reduce patient harm due to healthcare-associated infections (HAI), bloodstream infections (BSI) still loom large. However, after decades of intense scrutiny of – and success in preventing – central line-associated bloodstream infections (CLABSI), the time has come to broaden our perspective on preventable harm due to healthcare-associated BSI. In this commentary, we describe why stakeholders in quality and infection prevention should value a broader bacteremia event outcome, enumerate the advantages and potential pitfalls of a hospital-onset bacteremia/fungemia (HOB) measure, and share practical guidance on implementing quality improvement related to HOB events.

## BSI beyond CLABSI

With evidence-based toolkits and guidelines, acute care facilities achieved steady reductions in CLABSI rates prior to the COVID-19 pandemic.^1^ While much of this success is due to true reduction in adverse patient events, the impelling agents of financial penalties and publicly reported quality measures also nudge organizations toward reducing *reported* CLABSI without real harm reduction through source misattribution, intentional manipulation of reported HAI rates, and shifting BSI risk from central venous catheters to other forms of intravenous access.^2,3^

The transition from a focus on a parochial quality measure and “on paper” improvement with CLABSI to true and broader-scale harm reduction with HOB has precedent in the transition from ventilator-associated pneumonia to ventilator-associated events: a simpler and more objective definition, a broadening of the scope of harm, and new science demonstrating the preventability of these adverse events.^4,5^

The HOB definition is simple and objective: A bacterial or fungal pathogen from a blood culture specimen collected on the 4th calendar day of admission or later (where the date of admission to an inpatient location is day 1).^6^ A significant proportion of HOB events are attributable to a source that is not likely to be captured by traditional HAI surveillance and thus may not be included in current infection prevention toolkits. Early studies are establishing the preventability of this expansive proposed quality measure.^6^

## Implications of a changing BSI prevention paradigm

Hospital-onset bacteremia/fungemia provides an opportunity to re-evaluate current HAI prevention strategies given the scope and complexity of underlying etiologies and root causes. Quality teams will need to innovate and expand notions of “preventable” infections (Table 1).

**Table 1.** Expanding the paradigm of bloodstream infection (BSI) prevention for a hospital-onset bacteremia and fungemia metric

BSI reduction strategy	Rationale	Considerations for future intervention and innovation
Device and procedure-specific interventions	Significant proportion of BSI is not captured by the current narrow surveillance strategy Focus on device-specific risk may not capture overall risk	• Peripheral venous catheter-associated HOB prevention bundle • Expand BSI prevention beyond central venous catheters, urinary catheters, and limited surgeries with required surveillance of all devices and procedures. • Balance device-specific infection risk reduction against broader harm e.g., avoidance of urinary catheters should be one component of a larger strategy for appropriate urinary bladder management that also addresses risk of infection from urinary retention and suboptimal urinary drainage
Blood culture diagnostic stewardship	Blood culture collection intensity correlates withBSI rate, and a significant proportion are skin commensals	• Reduce high-intensity, low-value testing to reduce risk of blood culture contamination • Institute standardized evidence-based protocols for blood culture phlebotomy, including preference for percutaneous rather than catheter-drawn specimens • Engage palliative care services and critically assess diagnostic utilization at the end of life to ensure testing aligns with patient goals of care
Pathogen bioburden reduction	Microbial colonization is a key step in the pathogenesis of HOB	• Optimize use of chlorhexidine skin treatment in high-risk individuals • Enhance terminal room cleaning to mitigate environmental sources of multidrug-resistant organism (MDRO) • Reduce patient exposure to contaminated hospital water and plumbing • Advanced research on the utility of multi-drug resistant organism decolonization strategies such as selective digestive decontamination and fecal microbiota transplantation
Timely and effective source control of infection	Delays in effective source control are a risk for HOB as a complication of infections present on admission	• Conduct a critical evaluation of clinical management of intra-abdominal or gastrointestinal sources of infection, including the timing and approach to source control • Ensure timely surgical intervention for complex infections such as severe diabetic foot infections and infective endocarditis through multi-disciplinary care
Reducing hospital exposure and risks associated with prolonged stay	Time spent in the hospital is a critical exposure risk for HOB events Hospitalization rates and length of stay demonstrate the underlying quality of care within a health system	• Partner with patient safety teams on prevention of other hospital-acquired events that are sources of bacteremia e.g., decubitus ulceration, or that may prolong length of stay e.g., falls • Implement multi-disciplinary effort spanning pre-hospital and post-hospital settings to reduce hospital exposure: patient-centered medical home, hospital-at-home, discharge/throughput teams, leveraging health system integration to promote earlier transfer to affiliated rehabilitation and skilled nursing facilities
Systemic healthcare challenges: Staffing and social determinants	The landscape of healthcare staffing is antithetical to infection prevention efforts Racial and ethnic disparities exist within healthcare-associated infection rates and historically were not a priority for intervention	• Streamline infection prevention strategies to reduce task load on bedside teams • Strategic training of infection prevention nurse “champions” on units with high HOB incidence • Address social determinants including race/ethnicity, housing status, and substance use disorder that are sources of unconscious bias in care delivery

However, as with any other measure, there can be negative consequences and the potential to “game” the system, particularly if the measure is not supported by practice guidance and an implementation toolkit. Diagnostic stewardship will be necessary to prevent clinically unnecessary “surveillance” blood cultures on admission (to classify positive blood cultures as infections “present on admission”), and intentionally reduce blood culturing during hospital stay when clinically indicated (e.g., sepsis).

The HOB metric may work counter to antimicrobial stewardship by incentivizing clinically non-indicated antibiotic prophylaxis. This compunction may reflect an effort to reduce HOB events that do not appear preventable by current standards. To minimize this risk, HOB measurement and reporting should allow identification of potentially nonpreventable events, particularly HOB resulting from mucosal barrier injury or gastrointestinal translocation.^6^

## Developing a framework for evaluating HOB prevention

With the introduction of HOB as a novel quality metric, acute care hospitals across the country will find themselves collecting and reporting data on a larger number and a greater breadth of BSIs.^6^ This will include HOB events with sources of infection less familiar to Infection Prevention or Quality Improvement/Patient Safety teams. For the existing cadre of reported HAI metrics, there is robust, evidence-based guidance to prevent these infections.^7^ Thus, a practical approach to assessing HOB prevention that improves patient safety is needed to avoid the fate of previously failed healthcare quality measures, which led to unintentional and at times detrimental results.^8^

One common method of evaluating the care delivery processes associated with HAIs is the root cause analysis (RCA). Considering the potential scope of HOB scenarios, variability in the structure and completeness of RCAs would hinder the ability of front-line care teams to utilize the new HOB metric to its fullest extent in identifying gaps in patient care. Therefore, as part of a study supported by the Centers for Disease Control and Prevention Prevention Epicenters Program, we developed a framework to help hospitals evaluate HOB events and identify root causes for quality improvement.

Anticipating that evaluation of HOB events would widen the lens of preventability, we sought to develop a comprehensive, yet adaptable framework that could be employed for routine use in RCAs. We performed a literature review of existing frameworks for the evaluation and categorization of patient safety events and selected the Joint Commission on Accreditation of Healthcare Organizations (JCAHO) Patient Safety Event Taxonomy as the foundation for our framework.^9^ We then filtered the JCAHO taxonomy through the lens of infection prevention for HOB event evaluations and expanded the framework to meet the current challenges and priorities of healthcare delivery.

**Figure 1.**
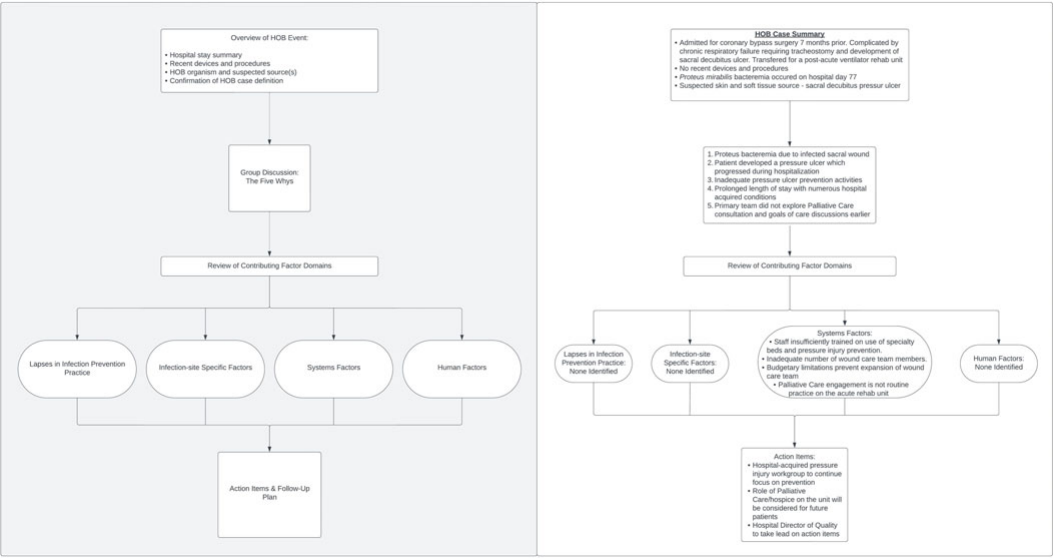
Flow diagram of a structured framework for the review of hospital-onset bacteremia and fungemia (HOB) cases (left). An example of a real-world HOB event as part of an ongoing prospective, multi-center study is provided to demonstrate the process and outcome of these case reviews (right).

The result of our work is a question-based, case review framework intended for multi-disciplinary engagement that greatly expands upon the current areas of focus for currently reportable HAIs (Figure 1). The primary domains of root causes explored within the framework are infection prevention factors, infection-site-specific factors, system factors, and human factors. Within each domain of the framework, cascading questions become more granular with affirmative responses, getting closer to the root causes and areas for improvement. Because the linkage between root causes – especially system-related – and patient outcomes is not always apparent to participating care teams, our case reviews exercise the “Five Whys,” an investigative technique to identify the root causes of events.^10^ The HOB event is established as a problem statement, to which the moderator asks “Why did this occur?,” and repeatedly does so as the layers beneath the HOB event are revealed. In this process, there is opportunity for participants to explore contributing factors and generate robust discussion even before the more directed portion of the framework begins.

In an ongoing multi-center study of the usability of HOB as a quality improvement metric, we are using this framework to discuss real-world HOB events with stakeholders. More expansive and exploratory, yet standardized, approaches such as this will be required to assess HOB preventability beyond what traditional device and procedure-related infection prevention toolkits offer.

## New challenges and new opportunities

Hospital-onset bacteremia and fungemia is a simplified BSI measure that pushes the boundaries of preventability, and as hospitals find themselves confronting unfamiliar root causes, new opportunities for harm prevention will be uncovered. The greatest benefit of an ambitious effort to reduce HOB rates may be its connectedness to other aspects of patient care, driving patient-centered quality improvement beyond the current scope of nosocomial infection prevention. It is critical, however, that infection prevention teams, hospital leaders, and public health agencies are prepared for this paradigm shift in HAI reporting to manifest the intended benefit and avoid gamesmanship. Development of standardized frameworks for HOB evaluation while in parallel identifying and investigating new strategies for prevention will be critical to a successful drive toward higher quality care.
